# Fast method of determining the ambient dose equivalent at a depth of 10 mm of gamma-neutron fields based on recombination methods

**DOI:** 10.1093/rpd/ncad016

**Published:** 2023-10-11

**Authors:** Michał Kuć

**Affiliations:** Radiological Metrology and Biomedical Physics Division, National Centre for Nuclear Research, Andrzeja Sołtana 7, 05-400 Otwock, Poland; Institute of Metrology and Biomedical Engineering, Warsaw University of Technology, Św. A. Boboli 8, 02-525 Warsaw, Poland

## Abstract

The subject of the work is the presentation of a new measurement algorithm to be used in dosimetry, based on recombination chambers and methods. The algorithm enables fast measurements of the ambient dose equivalent rate H^*^(10) with a time resolution of several seconds. In addition to significantly reducing the measurement time, the method allows for the automation of the measurement with continuous evaluation of the results quality. Operating principles are based on frequent charge measurements with precise determination of the measurement moment. With well-known charge and measurement time, the ionisation current and therefore H^*^(10) can be easily calculated. The time constant of such a system is close to zero and allows to shorten the measurement time dozens of times while improving the quality of measurement by analysing the collected charge course.

## Introduction

Unknown, mixed radiation fields are those in which there are more than one type of radiation components and in addition, the contribution of particles and/or their energy spectra are not known. In the context of radiation protection and biomedical engineering, gamma-neutron fields are the most common fields. Amongst the measurement methods of mixed radiation fields, a number of algorithms based on the initial gas recombination in the ionisation chamber can be distinguished^(1, 2)^. Detectors that use the phenomenon of initial recombination in gases are linear energy transfer-dependent detectors, called recombination chambers. The main areas of use of the recombination methods are: radiation protection at workplaces^(3)^, radiation protection during the conventional radiotherapy and hadron radiotherapy, in particular proton and neutron Boron Neutron Capture Therapy radiation therapies^(4–7)^, and material research^(8)^. Measurement set-up operating in the current mode (standard method) gives good results both in radiation fields with absorbed dose rates at the level of natural background and in ranges typical for radiotherapy. Despite the enormous advantages of recombination methods, there are problems that still limit the scope of their applicability. These include the most significant ones: high level of connection complexity and sensitivity to external conditions of the measuring system, duration of measurements, as well as the necessary interpretation of the results obtained by an experienced operator.

The subject of this research is to develop a new fast measurement algorithm based on recombination chambers—measurement set-up operating in the charge mode. The proposed algorithm allows for obtaining results no worse than with the standard method, but decreases significantly the acquisition time and improves the operational aspects of the measurement.

**Figure 1:**
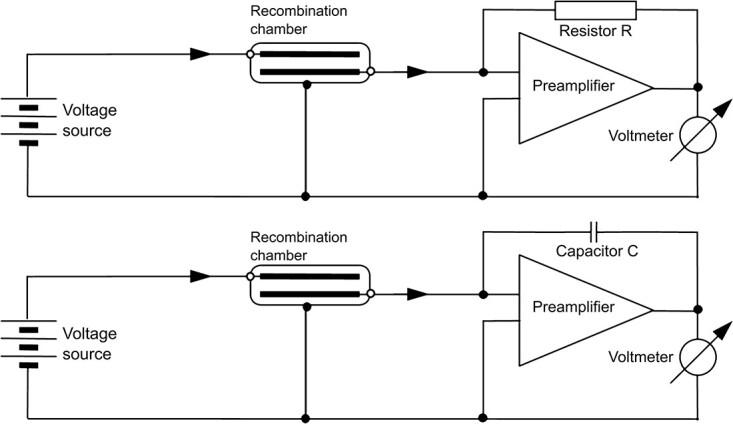
Electrical diagram of the measuring system based on the typical digital electrometer (e.g. Keithley 6517B) operating in current mode—standard method (top) and the charge mode—novel method (bottom).

## Materials and methods

A well-known recombination chamber of the REM-2 type was used in the study^(1–5, 7)^. It is an ambient dose equivalent rate H^*^(10) detector in fields of unknown composition and a wide energy spectrum of particles. The measurement of the H^*^(10) using REM-2 detector is carried out by:

- direct measurement of the ambient absorbed dose rate described in Equation (1).^(1)^
 
  (1) \begin{equation*} {D}^{\ast }(10)=\frac{I_S}{A} \end{equation*}
   
  - measurement of the ionisation current I_S_ for voltage approximately equal to saturation U_S_ with consideration of calibration coefficient A. Detector typically is calibrated in ^137^Cs or ^60^Co γ radiation source under saturation conditions;
- indirect measurement of the recombination radiation quality factor Q_R_ by comparing the I_S_ value with the ionisation current value I_R_ for recombination voltage U_R_. In radiological protection of unknown radiation fields, the best match of the REM-2 detector response to the radiation quality factor Q is for $R=4$^(1, 2, 9)^, then Q_4_ is described according to the Equation (2).

(2) \begin{equation*} {Q}_4=\frac{1-\frac{I_R}{I_s}}{0.04}; \end{equation*}

-
${H}^{\ast }(10)$ ^(1)^ is described by Equation (3).

(3) \begin{equation*} {H}^{\ast }(10)={D}^{\ast }(10)\cdotp{Q}_4 \end{equation*}

The Figure 1 shows the measuring systems based on electrometer Keithley K6517B type operating in the current and charge mode^(10)^. The difference between the systems is the use of a resistor R or a capacitor C in the feedback loop of the preamplifier. In both cases the current is determined by measuring the voltage at the preamplifier output. The methods of obtaining the current are described by Equations (4) and (5):

(1) in the first case the current $I(t)$ is directly proportional to the measured voltage $U(t)$ taking into account the time response of the system strongly dependent on the resistance R value and capacitance of the recombination chamber C*_rc_*,
  (4) \begin{equation*} I(t)=\frac{\mathrm{U}\left(\mathrm{t}\right)}{R\left(1-{e}^{-\frac{t}{R{C}_{rc}}}\right)} \end{equation*}
(2) in the second case, the current $I(t)$ is proportional to the voltage change over time $\frac{dU}{dt}$ and capacitance C.

(5) \begin{equation*} I(t)=C\frac{dU}{dt}=\frac{dQ}{dt} \end{equation*}

The most important difference between the two modes is the approximately zero time constant τ of the system operating in the charge mode. For each measuring modes, selection of R and C depends on the range of the I value. For the low value of ionisation current, the high value of R in the current mode must be chosen, and in this case the time constant $\tau = RC$ in current mode is high and makes the system response to changing conditions slow. In consequence, each measurement point with the current (standard) measurement method requires at least 120 seconds of system stabilisation after applying voltage and another 100 seconds of measurement.

The new algorithm implemented for the set-up operating in the charge mode uses the immediate response time caused by the time constant $\tau$ equal to zero. The new algorithm consists in measuring the course of the charge over time with the use of the internal electrometer samples buffer. Ionisation current for each measurement point was calculated according to the Equation (6) describing mean of the difference of the charge Q over time t with the sample buffer length n:


(6) \begin{equation*} I=\frac{\sum \frac{\varDelta Q}{\varDelta t}}{n-1} \end{equation*}

**Figure 2:**
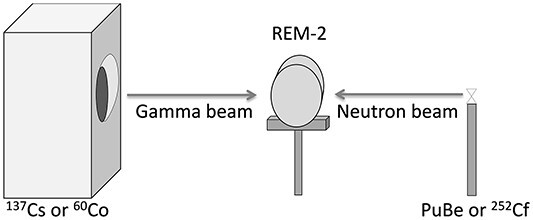
Experimental set-up. Neutron radiation sources were exposed manually, gamma radiation sources were exposed using remotely controlled irradiator, whereas the neutron source was exposed. Two measurement sessions followed one another directly.

The aim of the measurements was to compare the new measurement method with the standard one in terms of shortening the measurement time while maintaining the correctness of the obtained results. The new measurement algorithm was tested in gamma and neutron radiation fields with an ambient dose equivalent range from 200 μSv/h to 1 mSv/h. The experiment was carried out in the accredited laboratory (AP 070) at National Centre for Nuclear Research with the use of mixed neutron gamma fields. Before the measurements, the chamber was calibrated at close to saturation voltage U_S_ = 990 V with a ^137^Cs reference photon source. Two experimental sessions were conducted in different radiation sources configurations (listed in the header row of Table 1). The arrangement of the sources and the detector in the laboratory is shown in Figure 2:

**Table 1:** Mean m, standard deviation σ and the σ/m ratio of the absorbed dose rate D^*^(10), recombination radiation quality factor Q_4_ and ambient dose equivalent rate H^*^(10). Configurations in the first measurement session: ^239^PuBe, ^239^PuBe + ^60^Co, ^239^PuBe + ^137^Cs. Configurations in the second measurement session: ^252^Cf, ^252^Cf + ^137^Cs.

	^**239**^ **PuBe**			^**239**^ **PuBe +** ^**60**^**Co**			^**239**^ **PuBe +** ^**137**^**Cs**			^**252**^ **Cf**			^**252**^ **Cf +** ^**137**^**Cs**		
	**m**	**σ**	**σ/m**	**m**	**σ**	**σ/m**	**m**	**σ**	**σ/m**	**m**	**σ**	**σ/m**	**m**	**σ**	**σ/m**
D^*^(10) [μGy/h]	32.68	0.46	**1.42%**	53.42	0.44	**0.82%**	900.98	0.32	**0.04%**	31.76	0.47	**1.48%**	660.37	0.79	**0.12%**
**Q**_**4**_ **[Sv/Gy]**	6.68	0.41	**6.09%**	4.22	0.27	**6.42%**	1.12	0.01	**1.07%**	6.36	0.37	**5.80%**	1.21	0.04	**3.39%**
H^*^(10) [μSv/h]	218.40	15.77	**7.22%**	225.39	15.73	**6.98%**	1013.28	10.59	**1.05%**	202.23	13.95	**6.90%**	801.13	27.92	**3.48%**

Session 1: ^239^PuBe neutron source and/or ^60^Co or ^137^Cs γ radiation source,

Session 2: ^252^Cf neutron source and/or ^137^Cs γ radiation source.

## Results

**Figure 3:**
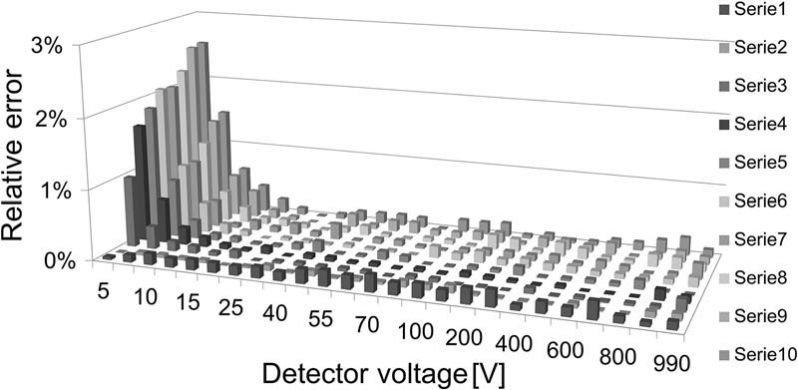
Relative error of the ionisation current measured with the new method to the standard method. Figure shows subsequent identical series of measurements. In total, 100 charge points collected for each voltage of which the first 30 points are ignored (Figure 5).

**Figure 4:**
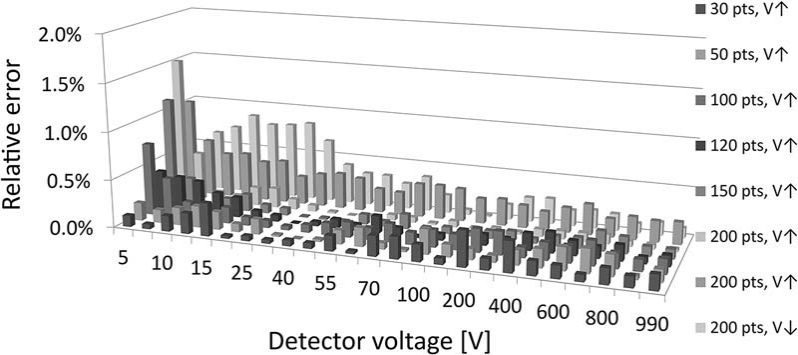
Relative error of the ionisation current measured with the new method to the standard method. Figure shows subsequent series of measurements; variable value of the number of points in the buffer; voltage ascending V↑ and descending V↓.

**Figure 5:**
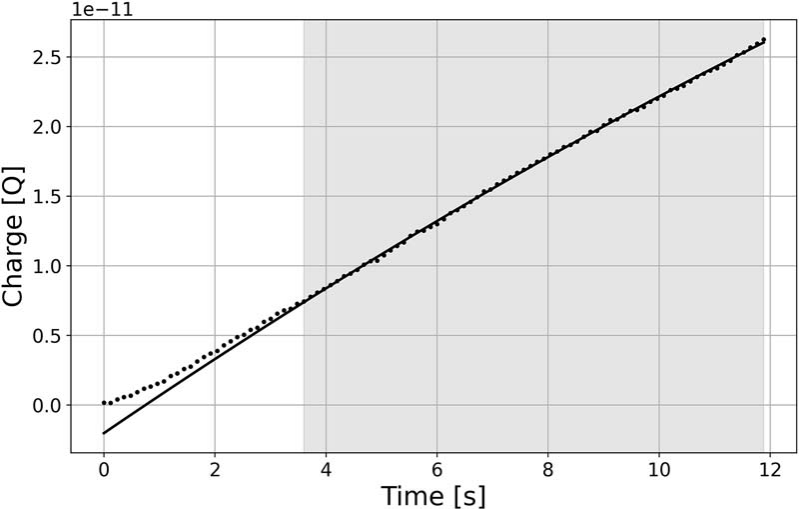
The time course of the charge (dots) in the example of Session 2 after the detector voltage changed from 990 to 55 V (from U_S_ to U_R_). Linear function has been fitted to the points in the shaded area. Non-linearity of the course of the charge over time due to the charging/discharging of the detector capacitance.

In Figures 3 and 4 relative error expressed as ratio of mean and standard deviation of the ionisation current measured with the new method to the standard method is shown. The detector was exposed to gamma radiation ^137^Cs, saturation current was about I_S_ ≈ 140 pA, and the ambient absorbed dose rate D^^*^^(10) ≈ 1.5 mGy/h. The duration of the measurement with the new method lasted from 5 to 24 s, depending on the number of samples in the buffer, which shortens the time from 9 to 44 times compared with the duration of the measurement with the standard method (220 s). Figure 3 shows the successive, identical series of measurements. In total, 100 charge samples collected for each voltage of which the first 30 were rejected as a non-linear (Figure 5). The graph shows an increase in the relative error for low voltages (below 12 V) for subsequent measurement series. It may be caused by insufficient stabilisation of the electrometer before the measurement series. The visible effect is not due to the measuring method itself but is the susceptibility of the electrometer.

Figure 4 shows the measurement results with a changing number of buffer samples with voltage ascending V↑ and descending V↓. For the presented results, the dependence of the relative error on the number of buffer samples is not visible. For 200 samples in the buffer, the measurement was repeated for the voltage change in ascending and descending order. The differences between these series may result from the detectors capacity charging/discharging effect presented in Figure 5.

Figure 5 shows the time course of the charge in the example of Session 2 with the ^252^Cf source after the detector voltage changed from 990 to 55 V (from U_S_ to U_R_). Linear function has been fitted to the points in the shaded area. The non-linearity in the first 2 seconds of the plot is caused by the charging or discharging of the detector capacitance as the voltage changes. Its contribution to the measurement error is the greater the smaller the value of the measured ionisation current. The results presented in the article take this effect into account by rejecting the first points.

**Figure 6:**
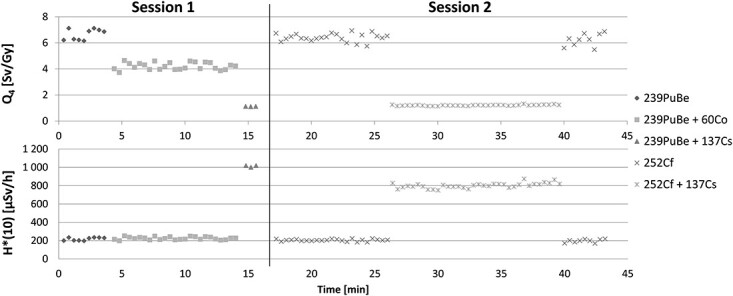
Q_4_ and H^*^(10) measurement results for two measurement sessions for different configurations of radioactive sources. The measurement is carried out in steps of 20 seconds.

In the Figure 6 measurements of Q_4_ and H^*^(10) for various configurations of radioactive sources are shown. H^*^(10) and Q_4_ measurements were performed approximately every 20 seconds. Table 1 presents the measured values: mean m, standard deviation σ and the ratio σ/m of the D^*^(10), Q_4_ and H^*^(10). The measurement relative uncertainty of the presented results D^*^(10), Q_4_ and H^*^(10) is the greater the lower the value of the measured equivalent of the absorbed dose is.

Measurements carried out in the past^(4–7)^ reveal research around medical accelerators, where the most advanced recombination methods are Q_4_ and H^*^(10) determination. The presented measurement algorithm, compared to the standard one, apart from shortening the duration of measurements, also allows for the implementation of previously unattainable tests. The desired dosimetric method to be used in such fields would be a recombination microdosimetric method (RMM)^(2, 11)^, which requires the implementation of full current–voltage characteristics detector—at least 50 voltage points. The duration of measurements for the RMM method in the standard mode is about 3 hours. Using the new algorithm, in the version presented in the article, such a characteristic can be achieved in less than 10 minutes.

## Conclusion

The new fast algorithm for measurements with recombination chambers operating in the charge mode was developed. It may increase the attractiveness and applicability of recombination methods in the field of radiation protection, because it allows the significant decrease of acquisition time, as well as improve the operational aspects of measurements.

## Data availability

The data that support the findings of this study are available on request from the corresponding author.

## References

[1] Golnik, N., Zielczynski, M., Bulski, W., Tulik, P. and Palko, T. Measurements of the neutron dose near a 15 mv medical linear accelerator. Radiat. Prot. Dosim. 126(1–4), 619–622 (2007). 10.1093/rpd/ncm125 17513292

[2] Golnik, N. Recombination chambers—do the old ideas remain useful? Radiat. Prot. Dosim. 180(1-4), 3–9 (2018). 10.1093/rpd/ncx279 29237076

[3] Ambrožová, I. et al. REFLECT – research flight of EURADOS and CRREAT: Intercomparison of various radiation dosimeters onboard aircraft. Radiat. Meas. 137, 106433 (2020).

[4] Wochnik, A. et al. Out-of-field doses for scanning proton radiotherapy of shallowly located paediatric tumours—a comparison of range shifter and 3D printed compensator. Phys. Med. Biol. 66(3), 035012 (2021). 33202399 10.1088/1361-6560/abcb1f

[5] Tulik, P., Tulik, M., Maciak, M., Golnik, N., Kabat, D., Byrski, T. and Lesiak, J. Investigation of secondary mixed radiation field around a medical linear accelerator. Radiat. Prot. Dosim. 180(1–4), 252–255 (2017). 10.1093/rpd/ncx199 29036647

[6] Zielczynski, M., Gryzinski, M. A., Golnik, N. and Tulik, P. Ionisation chamber containing boron as a neutron detector in medical accelerator fields. Radiat. Prot. Dosim. 126(1–4), 274–277 (2007). 10.1093/rpd/ncm057 17575294

[7] Zielczynski, M., Golnik, N., Gryzinski, M. A. and Tulik, P. The use of recombination chambers at radiation therapy facilities. Radiat. Meas. 45(10), 1472–1475 (2010).

[8] Domański, S., Gryziński, M. A., Maciak, M., Murawski, Ł., Tulik, P. and Tymińska, K. Experimental investigation on radiation shielding of high performance concrete for nuclear and radiotherapy facilities. Pol. J. Med. Phys. Eng. 22(2), 41–47 (2016).

[9] ICRP . 1990 recommendations of the international commission on radiological protection. ICRP publication 60. Ann. ICRP 21(1–3), 1–201 (1991). 2053748

[10] Keithley Instruments . Model 6517B Electrometer User’s Manual; 6517B-900-01 Rev. A/Jun. ( Cleveland, Ohio, U.S.A.: Keithley Instruments, Inc.) (2008).

[11] Golnik, N. Microdosimetry using a recombination chamber: method and applications. Radiat. Prot. Dosim. Keithley Instruments, Inc. 61(1–3), 125–128 (1995).

